# The CAMKK/AMPK Pathway Contributes to *Besnoitia besnoiti*-Induced NETosis in Bovine Polymorphonuclear Neutrophils

**DOI:** 10.3390/ijms25158442

**Published:** 2024-08-02

**Authors:** Iván Conejeros, Zahady D. Velásquez, Lisbeth Rojas-Barón, Gabriel Espinosa, Carlos Hermosilla, Anja Taubert

**Affiliations:** Institute of Parasitology, Justus Liebig University of Giessen, 35392 Giessen, Germany; zahady.velasquez@vetmed.uni-giessen.de (Z.D.V.); lisbeth.cecilia.rojas.baron@vetmed.uni-giessen.de (L.R.-B.); gabriel.rodolfo.espinosa.espinoza@vetmed.uni-giessen.de (G.E.); carlos.r.hermosilla@vetmed.uni-giessen.de (C.H.); anja.taubert@vetmed.uni-giessen.de (A.T.)

**Keywords:** *Besnoitia besnoiti*, PMN, NETs, cattle, apicomplexan, neutrophils, bovine, innate immunity

## Abstract

*Besnoitia besnoiti* is an obligate intracellular apicomplexan parasite and the causal agent of bovine besnoitiosis. Bovine besnoitiosis has a considerable economic impact in Africa and Asia due to reduced milk production, abortions, and bull infertility. In Europe, bovine besnoitiosis is classified as an emerging disease. Polymorphonuclear neutrophils (PMN) are one of the most abundant leukocytes in cattle blood and amongst the first immunological responders toward invading pathogens. In the case of *B. besnoiti*, bovine PMN produce reactive oxygen species (ROS), release neutrophil extracellular traps (NETs), and show increased autophagic activities upon exposure to tachyzoite stages. In that context, the general processes of NETosis and autophagy were previously reported as associated with AMP-activated protein kinase (AMPK) activation. Here, we study the role of AMPK in *B. besnoiti* tachyzoite-induced NET formation, thereby expanding the analysis to both upstream proteins, such as the calcium/calmodulin-dependent protein kinase kinase 2 (CAMKK), and downstream signaling and effector molecules, such as the autophagy-related proteins ULK-1 and Beclin-1. Current data revealed early AMPK activation (<30 min) in both *B. besnoiti*-exposed and AMPK activator (AICAR)-treated bovine PMN. This finding correlated with upstream responses on the level of CAMKK activation. Moreover, these reactions were accompanied by an augmented autophagic activity, as represented by enhanced expression of ULK-1 but not of Beclin-1. Referring to neutrophil effector functions, AICAR treatments induced both AMPK phosphorylation and NET formation, without affecting cell viability. In *B. besnoiti* tachyzoite-exposed PMN, AICAR treatments failed to affect oxidative responses, but led to enhanced NET formation, thereby indicating that AMPK and autophagic activation synergize with *B. besnoiti*-driven NETosis.

## 1. Introduction

Bovine besnoitiosis is classified as an emerging disease by the European Food Safety Authority (EFSA) and has important economic impact in Africa, especially in sub-Saharan countries, Asia, and Europe. Only a single report from South America is described. No reports from North America or Australia are known. The causal agent of bovine besnoitiosis is the apicomplexan, cyst-forming parasite species *Besnoitia besnoiti*. In the acute phase, *B. besnoiti*-infected cattle show pyrexia, lacrimation, photophobia, orchitis, vulvitis, vestibulitis, tachypnoea, tachycardia, and subcutaneous edema as main symptoms. During the chronic phase of the infection, the symptoms vary depending on the organ in which the parasitic cysts develop [1,2,3]. *B. besnoiti* cysts can be found on skin, sclera, vasculature, and non-intestinal mucous membrane, such as the reproductive tract of males and females. Symptoms include alopecia and scleroderma with skin thickening, and this alteration can cause improper milk if the udder skin is affected [3,4,5]. In bulls, chronic *B. besnoiti* infection may cause orchitis, affecting fertility [4]. Regarding transmission, *B. besnoiti* can be mechanically transmitted by haematophagous tabanids (*Tabanus* spp.), biting muscids, such as *Stomoxys calcitrans*, and bloodsucking arthropods. Typically, the herds are diagnosed by clinical inspection supplemented with serology tests. The role of transmission via mating remains unclear. To date, the natural transmission route and the definitive host of *B. besnoiti* remain unclear [4,5].

In *B. besnoiti*-infected cattle, immunological responses driven by tachyzoite stages include leukocyte infiltration in the dermis of affected animals [6]. Polymorphonuclear neutrophils (PMN) are amongst the first innate immune cell types responding against infection. PMN are an important part of the cellular response of the innate immune system, reacting to invasive pathogens, such as bacteria, viruses, fungi, and parasites. PMN-derived effector mechanisms include the secretion of cytokines and chemokines, phagocytosis, production of reactive oxygen species (ROS), and the release of neutrophil extracellular traps (NETs) [7,8,9]. NETs are composed of DNA, histones, and microbicidal peptides, such as neutrophil elastase, myeloperoxidase, and histones [9]. NETs limit pathogen spread in infected organisms by local trapping [10] and are released via a cell death process known as NETosis [11]. After initial PMN activation, neutrophil enzymes, such as neutrophil elastase (NE) and myeloperoxidase (MPO), translocate to the nucleus and fuse with chromatin. This induces nuclear pore formation and, finally, NET extrusion into the extracellular space occurs [12,13]. NET release is induced by several protozoan parasites, such as *Eimeria bovis* [14], *Eimeria arloingi* [15], *Toxoplasma gondii* [16,17], *Cryptosporidium parvum* [18], *Neospora caninum* [19], *Trypanosoma brucei brucei* [20], and *B. besnoiti* [21,22,23,24].

Notably, *B. besnoiti* tachyzoite-driven NETosis seems stage-independent since both tachyzoites and bradyzoites were proven to trigger NET formation in bovine PMN [25]. Metabolic responses of *B. besnoiti* tachyzoite-exposed bovine PMN include enhanced catabolism of glucose and serine, accompanied by increased glutamate production [26]. Secondary metabolites in *B. besnoiti* tachyzoite-exposed PMN appear pivotal for NET formation since chemical inhibition of lactate release, pyruvate dehydrogenase, α-ketoglutarate dehydrogenase, and transketolase significantly affect cell-free NET formation, thereby indicating a key role of pyruvate- and lactate-mediated metabolic pathways in this effector mechanism. Interestingly, tachyzoite-induced NET formation is also significantly blocked by treatments with oligomycin A (inhibitor of ATP synthase) and NF449 (purinergic receptor P2X1 inhibitor), suggesting a key role of ATP-related responses in tachyzoite-exposed PMN [26]. The latter inhibitor, NF449, also impairs PMN clustering around *B. besnoiti* tachyzoites [24], a phenomenon that eventually represents an early event of *B. besnoiti*-triggered NET formation. Consequently, it is compelling to hypothesize that *B. besnoiti*-induced NET formation is tightly regulated by signaling pathways in the early phase (<30 min) of the PMN-tachyzoite interaction. In this regard, recent studies on signaling cascades upstream of *B. besnoiti* tachyzoite-driven NET extrusion have identified the phosphorylation of AMP-activated kinase α (AMPKα) as an important player in the regulation of cellular processes, such as neutrophil glycolytic metabolism, and stated a positive correlation between autophagy-positive (LC3B^+^-positive) PMN and NET formation [23]. Notably, NETosis and autophagy are described as interconnected processes in PMA-induced, ROS-dependent NET formation in human PMN [27,28]. Autophagy represents an essential intracellular degradation process that recycles cell components, such as proteins and organelles, and that proved essential in cellular responses to stress events [29]. Neutrophil autophagy is pivotal and interconnected with PMN functions and can prime PMN to undergo NET formation [23,27,28,29]. Besides other molecules, autophagy is regulated by the metabolic sensor molecule AMPKα and by the mechanistic target of rapamycin (mTOR) [30].

AMPK is well known for its cellular metabolic sensor functions. It is a serine/threonine protein kinase consisting of three heterogenic subunits: a catalytic α-subunit as well as regulatory β- and γ-subunits, which directly bind AMP. AMPK regulates the balance between catabolism and anabolism in response to changes in the cellular metabolic status [31]. This effect may either result from the inhibition of ATP generation (e.g., by hypoxia, glucose deprivation, heat shock, and a reduction in mitochondrial oxidative phosphorylation), or from increased ATP consumption, as occurring during exercise or in cases of cellular metabolism upregulation [32]. Therefore, AMPK participates in the activation of ATP-generating pathways involving uptake and oxidation of glucose and fatty acids and, furthermore, inhibits intracellular events that consume ATP and are not critical for the short-term survival of a cell. There is increasing evidence that AMPK also participates in modulating acute inflammatory reactions and plays a major role in regulating PMN functions [32,33]. In mice, AMPK activation enhances PMN chemotaxis and bacterial killing in vitro and in vivo and counteracts chemotaxis inhibition induced by LPS (≥30 ng/mL). Accordingly, chemical blockage of AMPK activation diminishes neutrophil chemotaxis [34]. On a mechanistic level, the pharmacological activation of AMPK by aminoimidazole carboxamide ribonucleotide (AICAR) drives actin polymerization, formation of PMN edges, phagocytosis, and bacterial killing in murine PMN [32,34,35]. Conversely, in human PMN, AMPK shows inhibitory effects on fMLP- and PMA-induced ROS production [36].

The current work aimed to study the role of AMPK activation and autophagy in *B. besnoiti* tachyzoite-exposed bovine PMN and to evaluate the effect of pharmacological AMPK activation on *B. besnoiti* tachyzoite-induced neutrophil oxidative responses and NET formation.

## 2. Results

### 2.1. PMN Exposed to B. besnoiti Tachyzoites Induces AMPK Phosphorylation

**Figure 1:**
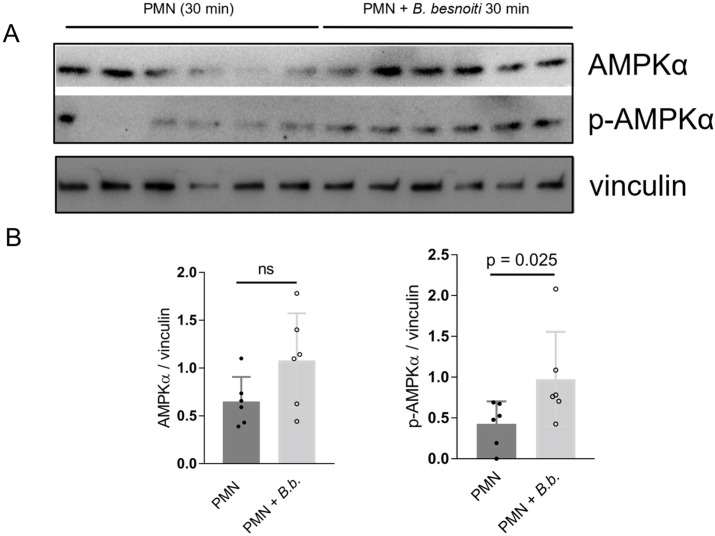
*B. besnoiti* tachyzoite exposure induces AMPK phosphorylation in bovine PMN. Bovine PMN isolated from peripheral blood from 6 different animals (*n* = 6) were exposed to *B. besnoiti* tachyzoites at a 1:6 PMN:*B. besnoiti* tachyzoites ratio. After 30 min of co-incubation, protein extracts were generated from PMN and tested for AMPK and p-AMPK expression by Western blotting. The expression of vinculin was used as an internal reference protein. (**A**) Western blot. (**B**) Densitometric analysis of protein bands for AMPK and p-AMPK. Bars in the graph represent mean ± SD. *p*-values were calculated by applying the Mann–Whitney test.

A previous report demonstrated AMPK phosphorylation as an early event of PMN–parasite interactions since it occurred already 10 min after incubation while being sustained for up to 30 min of co-incubation of bovine PMN with *B. besnoiti* tachyzoites [23]. Here, we confirmed this observation at the level of Western-blotting-based analyses of protein extracts originating from *B. besnoiti*-exposed PMN (Figure 1A). Hence, pAMPK but not AMPK revealed significantly enhanced expression at 30 min of co-culture (Figure 1B, parasite-exposed PMN vs. negative control condition: *p* = 0.025).

**Figure 2:**
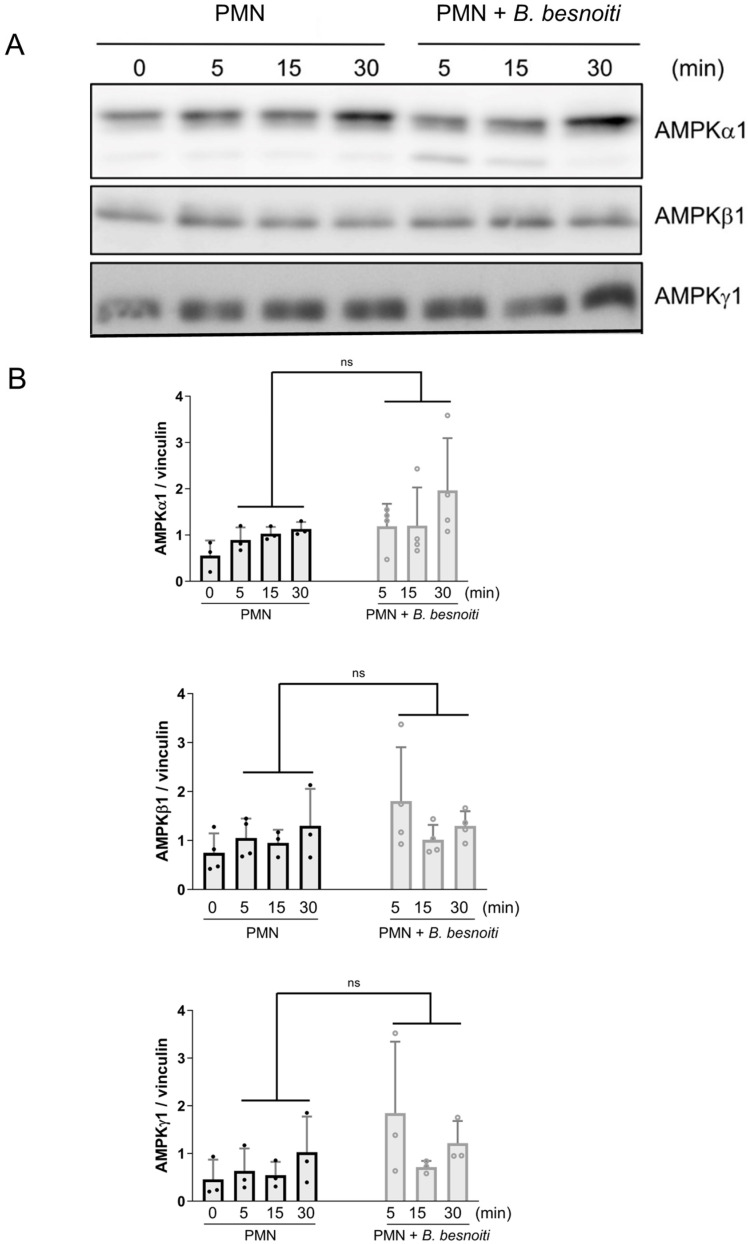
Analysis of AMPK catalytic and regulatory subunits in *B. besnoiti*-exposed bovine PMN. Bovine PMN isolated from peripheral blood from 4 different animals (*n* = 4) were exposed to *B. besnoiti* tachyzoites at a 1:6 PMN:*B. besnoiti* tachyzoites ratio. At 0–30 min of incubation, protein extracts were prepared from PMN and tested for AMPKα1, AMPKβ1, and AMPKγ1 expression by Western blotting. Vinculin was used as an internal reference protein. (**A**) Western blot. (**B**) Densitometric analysis of protein bands for AMPKα1, AMPKβ1, and AMPKγ1 at 0, 5, 15, and 30 min of PMN–tachyzoite coincubation. Bars in the graphs represent mean ± SD. *p*-values were calculated by unpaired, two-tailed *t*-tests, comparing control PMN vs. PMN incubated with *B. besnoiti* tachyzoites at each time point. ns means not significant.

Moreover, the catalytic subunit AMPKα1 revealed moderately but statistically insignificant (*p* = 0.110) upregulation in tachyzoite-exposed PMN (Figure 2A,B). Overall, no changes were observed for the regulatory subunits AMPKβ1 and AMPKγ1 (Figure 2A,B).

### 2.2. Tachyzoite Exposure Drives Significant CAMKK Upregulation and Phosphorylation in PMN

**Figure 3:**
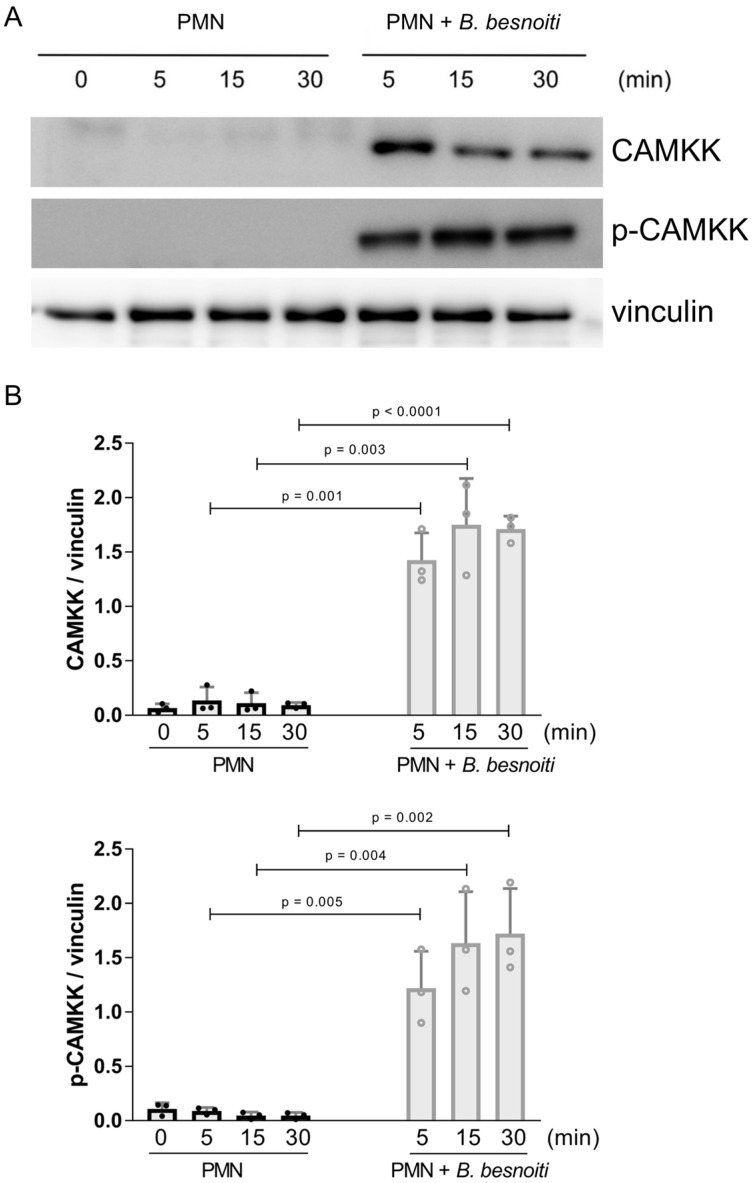
*B. besnoiti* tachyzoite exposure induces CAMKK expression and phosphorylation in bovine PMN. Bovine PMN isolated from peripheral blood from three different animals (*n* = 3) were exposed to *B. besnoiti* tachyzoites at a 1:6 PMN:*B. besnoiti* tachyzoites ratio. At 0–30 min of incubation, protein extracts were generated from PMN and tested for CAMKK and p-CAMKK expression by Western blotting. The expression of vinculin was used as an internal reference protein. (**A**) Western blot. (**B**) Densitometric analysis of protein bands for CAMKK and p-CAMKK at 0, 5, 15, and 30 min of PMN–tachyzoite incubation. Bars in the graph represent mean ± SD. *p*-values were calculated by unpaired, two-tailed *t*-tests, comparing control PMN vs. PMN incubated with *B. besnoiti* tachyzoites at each time point.

Considering that AMPK activity is mainly regulated upstream by CAMKK (besides other regulators and signaling pathways), here, we evaluated the expression and phosphorylation status of CAMKK at 5, 15, and 30 min of PMN exposure to *B. besnoiti* tachyzoites (Figure 3). PMN incubated in plain RPMI-1640 medium were used for the negative controls (Figure 3A). Densitometric analysis of respective protein bands in Western blots (Figure 3B) indicated that both phosphorylated and non-phosphorylated CAMKK were significantly upregulated in PMN immediately after tachyzoite encounter (from 5 min of exposure onwards; stimulated vs. control PMN at 5, 15, and 30 min: all *p* < 0.05), thereby indicating a highly sustained activation of CAMKK.

### 2.3. ULK1 Expression Is Upregulated in Tachyzoite-Exposed PMN

**Figure 4:**
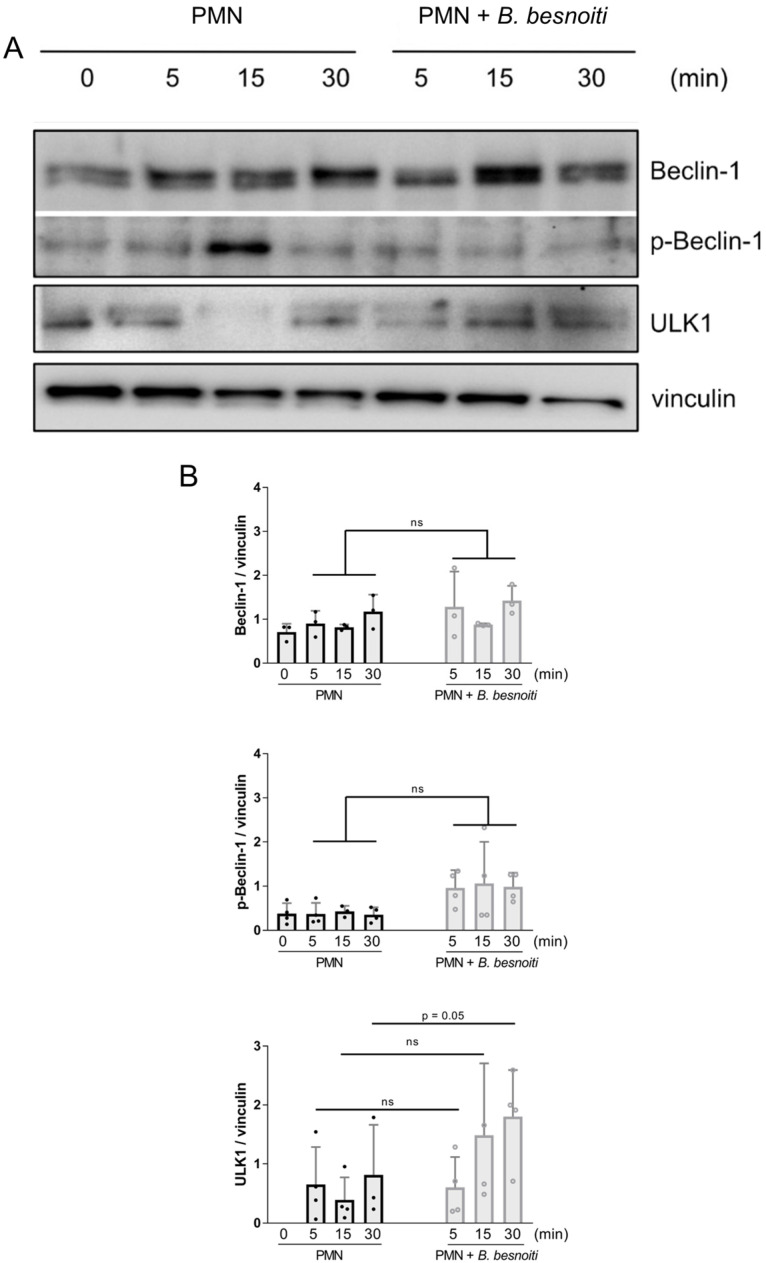
*B. besnoiti* tachyzoite exposure induces ULK-1 expression in bovine PMN. Bovine PMN isolated from peripheral blood from three different animals (*n* = 3) were exposed to *B. besnoiti* tachyzoites at a 1:6 PMN:*B. besnoiti* tachyzoites ratio. After 0–30 min of incubation, protein extracts were generated from PMN and tested for Beclin-1, p-Beclin-1, and ULK1 expression by Western blotting. The expression of vinculin was used as an internal reference protein. (**A**) Western blot. (**B**) Densitometric analysis of protein bands for Beclin-1, p-Beclin-1, and ULK1 at 0, 5, 15, and 30 min of PMN–tachyzoite incubation. Bars in the graph represent mean ± SD. *p*-values were calculated by unpaired, two-tailed *t*-tests, comparing control PMN vs. PMN incubated with *B. besnoiti* tachyzoites at each time point. ns means not significant.

Since PMN exposure to *B. besnoiti* tachyzoites induced LC3B-II expression in bovine PMN [23], here, we also studied early autophagic processes induced by tachyzoite encounter in more detail (Figure 4). Therefore, early expression profiles of the autophagy-related molecules Beclin-1, p-Beclin-1, and ULK1 were analyzed at 5, 15, and 30 min of bovine PMN-tachyzoite co-cultures (Figure 4A). Overall, out of these molecules, ULK-1 was exclusively and significantly upregulated after 30 min of co-incubation (Figure 4B; parasite-exposed PMN vs. non-exposed controls: *p* = 0.02), thereby indicating that parasite exposure indeed induces autophagosome biogenesis in bovine PMN.

### 2.4. AICAR Treatments Trigger AMPK Phosphorylation in Bovine PMN

**Figure 5:**
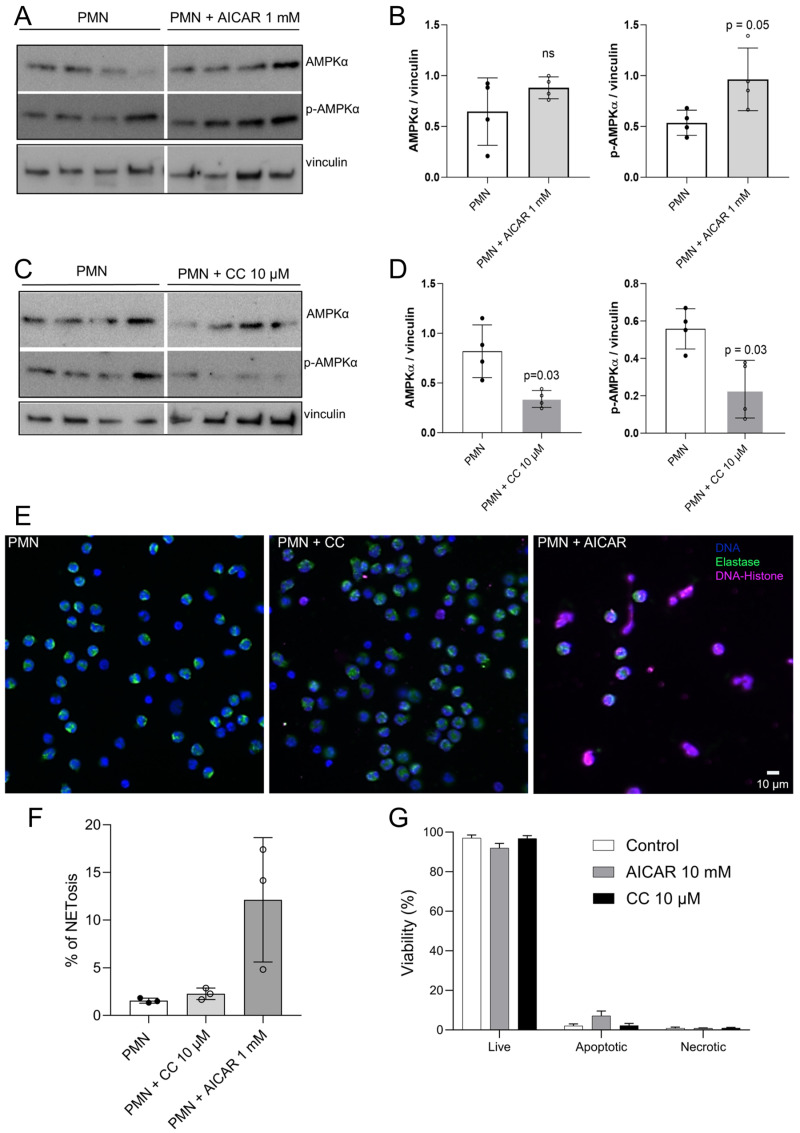
AICAR treatments drive AMPK phosphorylation and NET formation in bovine PMN. (**A**–**D**) Bovine PMN isolated from peripheral blood from 4 different animals (*n* = 4) were treated with AICAR (10 mM) or CC (10 µM) for 30 min. Thereafter, protein extracts were generated from PMN and tested for AMPKα and p-AMPKα expression by Western blotting. The expression of vinculin was used as an internal reference protein. (**A**,**C**) Western blot of AICAR-treated (**A**) or CC-treated (**C**) PMN. (**B**,**D**) Densitometric analysis of protein bands for AMPKα and p-AMPKα at 30 min of AICAR (**B**) or CC (**D**) treatments. Bars in the graph represents mean ± SD. *p*-values were calculated by applying the Mann–Whitney test. (**E**,**F**) Immunofluorescence images showing DNA (DAPI, blue), neutrophil elastase (NE, green), and DNA-histone complexes (magenta) in AICAR-treated bovine PMN (**E**). The percentage of NET-releasing PMN was calculated by a semi-automatic quantification method via image analysis (Image J, Fiji version) and is represented as a bar graph, showing mean ± SD (**F**). Viability of AICAR- and CC-treated bovine PMN, as evaluated by flow cytometry based on Annexin-V and propidium-iodide-positive staining. The percentages of live, apoptotic, and necrotic PMN are presented in the bar graphs (**G**). Bars in the graph represent mean ± SD.

To our best knowledge, no data are currently available on the metabolic efficacy of pharmacological AMPK activators (e.g., AICAR) or inhibitors (e.g., compound C; CC) in the bovine system. Therefore, based on published compound concentrations, here, we tested the effects of 1 mM AICAR and 10 µM CC treatments on AMPK phosphorylation in bovine PMN (Figure 5). As expected, AICAR treatments indeed induced AMPK phosphorylation in bovine PMN (Figure 5A,B; treated vs. control PMN: *p* = 0.05). Moreover, 10 µM CC treatments significantly reduced both AMPKα and p-AMPKα expression in bovine PMN (Figure 5C,D; treated vs. control PMN: *p* = 0.03). At the neutrophil functional level, AICAR treatments significantly affected neutrophil effector mechanisms by upregulating NET formation at 4 h of treatment (Figure 5E,F), thereby once more underlining the key role of AMPK phosphorylation for effective NETosis. As expected, CC treatments—leading to downregulation of AMPK phosphorylation—failed to affect NETosis. Importantly, neither AICAR nor CC treatments significantly affected PMN viability since the proportion of PMN experiencing apoptosis or necrosis was not changed by these treatments (Figure 5G).

### 2.5. AICAR Treatments Induce Metabolic Responses in Bovine PMN but Do Not Synergize with B. besnoiti-Induced OCR and ECAR

**Figure 6:**
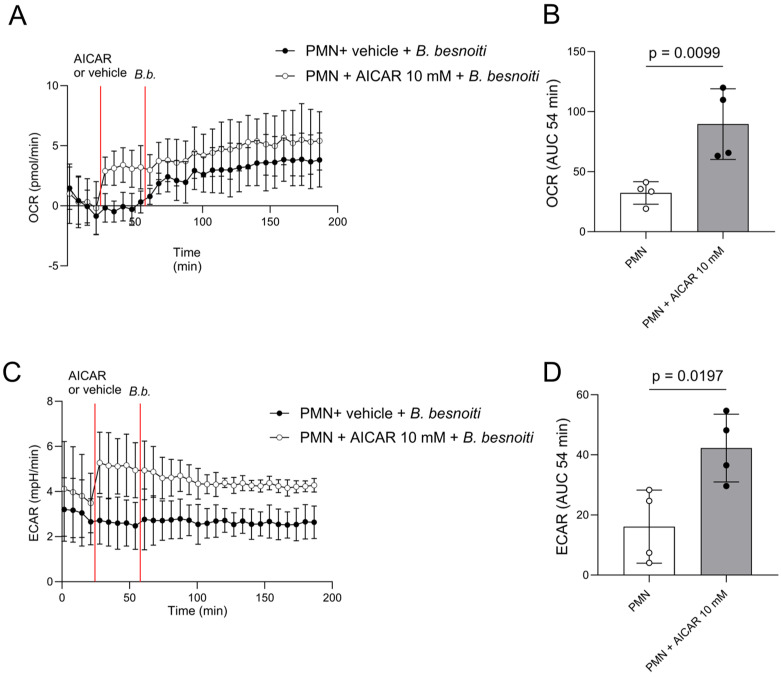
AICAR treatments induce oxygen consumption (OCR) and extracellular acidification (ECAR) rates in bovine PMN. Bovine PMN isolated from peripheral blood from 4 different animals (*n* = 4) were pretreated with AICAR or vehicle and then exposed to *B. besnoiti* tachyzoites at a 1:6 PMN:*B. besnoiti* tachyzoites ratio at 54 min. The effects of 10 mM AICAR treatments on tachyzoite-induced PMN responses were evaluated by Seahorse technology. AICAR treatments (white circles registry) induced both OCR (**A**,**B**) and ECAR (**C**,**D**), but failed to enhance *B. besnoiti*-induced OCR (**B**,**D**). The AUC of the registries after the baseline and before *B. besnoiti* tachyzoite supplementation was calculated to evaluate the effect of AICAR alone on OCR and ECAR. The bars represent mean ± SD. *p*-values were calculated by the Mann–Whitney test.

Considering that AMPK regulates the energetic status of a cell by sensing intracellular AMP concentrations and by adapting ATP synthesis in response to current cellular needs, we furthermore evaluated the effects of AICAR treatments on oxygen consumption rates (OCR; Figure 6A,B). OCR reflects neutrophil oxidative responses due to oxidative burst activity by assessing NADPHOX-related oxygen consumption, but also mirrors respiratory mitochondrial activity. Besides OCR, extracellular acidification rates (ECAR) were evaluated in AICAR-treated bovine PMN (Figure 6C,D). In general, ECAR levels were increased when cells shifted to a glycolytic state, based on production and release of lactate as a product of glycolysis. Current analyses revealed that AICAR treatments induced a significant rise in both OCR and ECAR in bovine PMN (Figure 6B,D, see the timeframe of 24–53 min). However, AICAR treatments failed to alter *B. besnoiti* tachyzoite-driven metabolic PMN responses (Figure 6A,C, white circles) and instead maintained the metabolic active status of PMN. The effect of AICAR alone on bovine PMN was analyzed after AICAR injection but before *B. besnoiti* tachyzoite supplementation (Figure 6B,D). The AUC analyses showed that OCR (treated vs. untreated PMN: *p* = 0.0099) and ECAR (treated vs. untreated PMN: *p* = 0.0197) were significantly increased in AICAR-treated bovine PMN.

### 2.6. Pharmacological AMPK Activation Boosts B. besnoiti Tachyzoite-Induced NET Formation

**Figure 7:**
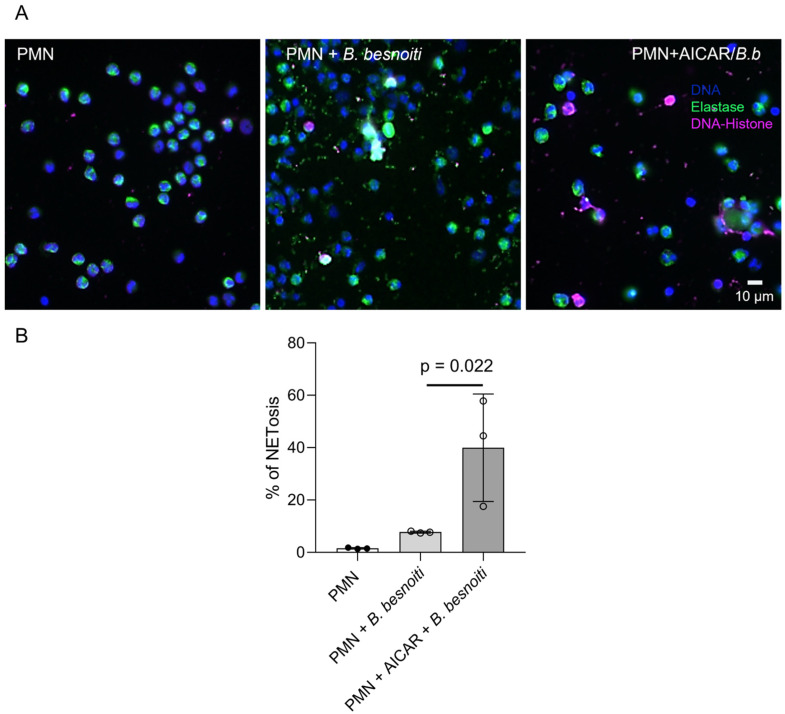
AICAR treatments enhance *B. besnoiti* tachyzoite-induced NET formation. Here, 2.5 × 10^5^ bovine PMN isolated from peripheral blood from three different animals (*n* = 3) were pretreated with 1 mM of AICAR or plain medium for 30 min before exposure for 4 h to *B. besnoiti* tachyzoites at a 1:6 PMN:*B. besnoiti* tachyzoites ratio. (**A**) Fixed samples were immunostained for neutrophil elastase (NE, green) and DNA-histone complex (magenta). DNA was stained with DAPI (**A**, upper row). (**B**) The percentage of NET-releasing cells was determined by a semi-automatic quantification method via image analysis (Image J, Fiji version). Bars in the graph represent mean ± SD. *p*-values were calculated by applying the Kruskal–Wallis test.

To further confirm the role of AMPK in the process of early NETosis, here, we assessed the additive effect of AICAR pretreatments on tachyzoite-driven NET formation (Figure 7). Therefore, bovine PMN were either exposed to *B. besnoiti* tachyzoites or additionally treated with AICAR. NET formation was evaluated by immunofluorescence and posterior image analyses using a semi-automatic method for NET quantification (Figure 7B) [37]. Current data show that AICAR treatments drive additive effects on NETosis since *B. besnoiti* tachyzoite-driven NET formation was significantly enhanced by AICAR supplementation when compared to plain parasite-mediated NET extrusion (Figure 7B; AICAR + tachyzoite-treated PMN vs. tachyzoite-exposed PMN: *p* = 0.022), thereby underlining the key role of AMPK activation in *B. besnoiti*-induced NET formation.

## 3. Discussion

In the present study, we added new data on the pivotal role of AMPK signaling in *B. besnoiti* tachyzoite-induced bovine PMN activation, as detected on the level of PMN energetic state analysis and NET formation. Increasing evidence showed that NET formation represents a conserved mechanism among multiple kingdoms [38,39]. Referring to protozoan parasites, NETosis is triggered by both extracellular parasites, such as *Trypanosoma brucei brucei* [20], *Trypanosoma cruzi* [40,41], or *Entamoeba histolytica* [42,43], and intracellular parasites, such as *T. gondii*, *E. bovis*, *C. parvum*, *N. caninum*, and *B. besnoiti* [14,16,17,19,22,23,24,44].

Meanwhile, several aspects of *B. besnoiti*-driven NETosis have been elucidated: *B. besnoiti* tachyzoites induce bovine NETs in a time- and dose-dependent manner [22] and, importantly, the events that drive NET formation seem tightly correlated with increased NE and MPO activity in addition to enhanced ROS production [22]. Extracellular DNA release from bovine PMN in response to *B. besnoiti* tachyzoites occurred irrespective of tachyzoite pretreatments (UV-attenuation, heat inactivation, and homogenization), despite varying in amplitude [22]. In the case of vital tachyzoites, their capacity to infect an endothelial cell monolayer was decreased after co-incubation with PMN [22]. The decreased infection capacity was restored after treatment with DNAse, indicating a major contribution of NETs to this effect [22]. Moreover, *B. besnoiti*-induced NET formation depends on P2X1 purinergic receptors, concomitant with increased PMN clustering and autophagy. The metabolic requirements of *B. besnoiti*-induced NET formation are mainly related to pyruvate- and lactate-mediated catabolic pathways and ATP availability [24,26]. *B. besnoiti*-driven NET formation proved stage-independent since bradyzoites recovered from skin cysts of infected animals also induced NETs and increased PMN autophagy [25]. Notably, primary bovine monocytes also formed extracellular traps (METs) when exposed to *B. besnoiti* tachyzoites [45], thereby emphasizing the general capacity of these parasitic stages to induce this innate effector mechanism and enhancing the likelihood of its occurrence during bovine besnoitiosis in vivo. Referring to *B. besnoiti* infection in cattle, the blood PMN counts typically decreased after acute infection, thereby suggesting margination and tissue infiltration as the most plausible causes [6]. Under physiological flow conditions, *B. besnoiti*-infected primary bovine endothelium showed increased expression of adhesion proteins, such as VCAM-1, P-selectin, ICAM-1, and E-selectin, thereby resulting in increased adhesion of bovine PMN. Also under physiological flow conditions, PMN induced endothelial damage on *B. besnoiti*-infected endothelium, an effect related to NET formation and to the presence of histone 2A (H2A) as one of the main components of NETs [21]. Notably, *B. besnoiti*-infected endothelium induces NETs as well [23]. Since *B. besnoiti*-induced bovine NET formation was extensively characterized in recent years, and due to the importance of *B. besnoiti* infection in cattle, here, we intended to add new aspects to the fundamental understanding of the NETotic process in pathogenesis of disease by analyzing the role of the AMPK-related signaling pathway in *B. besnoiti*-induced NETosis.

AMPKα is a metabolic master regulator in eukaryotes with a high impact on several important cellular mechanisms. AMPKα activation is initiated by changes in the metabolic status, mainly resulting from the inhibition of ATP generation during hypoxia, glucose deprivation, or increased ATP consumption [32]. Previous observations in PMN showed AMPK activation to diminish PMA-mediated ROS production in human PMN [36], but to enhance PMN chemotaxis, bacterial killing, MMP-8 secretion, and phagocytosis [34,46]. In the bovine system, neutrophil AMPK is activated by β-hydroxybutyrate and hydroxycarboxylic acid receptor 2 (HCA2) agonists [47], and AMPK is related to increased autophagy in estrogen (17β-estradiol, E2)-treated PMN in low-glucose (2.5 mM) settings, leading to increased LC3, ATG5, and Beclin-1 expression [48]. Moreover, AMPK induction promotes autophagy by directly activating ULK1—which is an enzyme downstream of mTOR—during autophagosome formation [49]. Since exposure to *B. besnoiti* tachyzoites was proven to induce AMPK phosphorylation in bovine PMN after 30 min of PMN/tachyzoites incubation and a positive correlation with LC3B expression was observed [23], here, we studied responses of specific AMPK subunits and downstream autophagy-related proteins, such as Beclin-1 and ULK-1. The current data showed that exposure of PMN to *B. besnoiti* tachyzoites indeed induced AMPKα activation in a time-dependent manner, occurring as early as 5 min after exposure. During the first 30 min of interaction, there were no changes in the regulatory subunits AMPKβ and AMPKγ, resulting in a sustained activation. However, one limitation of the current study is that we did not include the AMPKα2 subunit, which was also demonstrated to be upregulated in activated PMN, especially in a hypoxia-related inflammation context [50]. Interestingly, AMPK is also involved in fluorine-induced ROS and NETs in carps, an effect that is inhibited by compound C [51], indicating that AMPK-controlled NET release is an ancient and conserved mechanism.

When referring to the AMPK-related signaling pathway, CAMKK is a prominent enzyme located upstream of AMPK phosphorylation. CAMKK is activated by a rise in the intracellular calcium concentration (Ca^2+^). It is expressed in human PMN and participates in the regulation of neutrophil respiratory burst and chemoattractant-induced PMN migration [52]. However, there is almost no information on CAMKK expression or phosphorylation in bovine neutrophils. One report studied CAMKK responses at the transcriptomic level in PMN of cows experiencing subclinical hypocalcemia and found that calcium binding- and calcium-signaling-related proteins showed decreased expression in this scenario [53]. Current data showed that both CAMKK expression and phosphorylation were highly upregulated in bovine PMN (≥ 6-fold increase) after exposure to *B. besnoiti* tachyzoites. Considering that this response was uniform in all PMN donors, occurred in a fast manner, as early as 5 min post-interaction, and remained sustained over 30 min, it was most probably linked to and consistent with the parasite-driven AMPK activation mentioned above.

Autophagy is a physiological process, which maintains homeostasis or normal cell function by protein degradation and turnover of destroyed cell organelles for new cell formation, especially in response to cellular stress [54]. Furthermore, autophagy plays a pivotal role in regulating early innate leukocyte-associated effector mechanisms against pathogens, such as phagocytosis [55], cytokine secretion [56], and NET formation [27]. Inflammatory inducers of autophagy include PAMPs, TLRs, TLR adaptors, ROS, NOD-like receptors, and AMPK [23,27,55]. In this regard, AMPK-related signaling plays a key role in NET formation via the regulation of autophagy pathways [23,27,28]. AMPK promotes autophagy, among other signaling cascades, by directly activating the pre-initiation complex ULK1 through phosphorylation [57]. In the current study, *B. besnoiti* tachyzoite exposure to bovine PMN induced neutrophil ULK-1 upregulation, thereby temporally coinciding with AMPK activation. In contrast, no changes were observed for Beclin-1 protein expression, indicating that a Beclin-1-independent autophagy pathway operates in *B. besnoiti*-exposed PMN. Since ULK-1 but not Beclin-1 was shown to be involved in phagocytosis-related autophagy, current data may indicate a similar mechanism to be driven by *B. besnoiti* tachyzoite exposure [23,58]. Thus, these responses seem directly linked and complementary, which is in line with a previous report on the presence of increased numbers of LC3B-positive autophagosomes in *B. besnoiti*-exposed PMN [23]. Since LC3B induction was also reported as associated with enhanced phagocytosis [59], both effector mechanisms are most probably related and interconnected in *B. besnoiti*-exposed PMN.

In general, it is reported that AICAR treatments improved bacterial killing and phagocytosis [34] but inhibits PMN apoptosis [60]. Compound C is used as an AMPK inhibitor, but considered non-specific. In bone-marrow-derived neutrophil-like cells, compound C inhibited chemotaxis in a dose-dependent manner [34]. In human PMN, it inhibited the MMP-8 release induced by conditioned media from monocytes infected by *Mycobacterium tuberculosis* [46]. To our knowledge, this is the first report on the use of compound C for bovine PMN treatments, showing an inhibition of both AMPK and p-AMPK protein expression, without affecting PMNs’ viability. Unexpectedly, compound C treatments failed to considerably affect NET formation in the current study.

To further study the role of AMPK activity in *B. besnoiti*-induced PMN activation, we first showed that the pharmacological AMPK activator, AICAR, indeed worked in the bovine system and promoted AMPK phosphorylation in bovine PMN. This is coherent with results in human PMN [36]. In addition, here, we demonstrated that plain AICAR treatments induced NET formation and triggered both mitochondrial and glycolytic responses in bovine PMN. Notably, AICAR treatments also resulted in additive effects referring to *B. besnoiti* tachyzoite-driven NET formation. Moreover, AICAR induced moderate oxidative responses in PMN, which may support the idea of NOX-dependent NET formation for the bovine system. In contrast to current data, AICAR treatments reduced PMN-derived ROS production in response to PMA stimulation and failed to induce H_2_O_2_ production in the human system, even though this effect proved dependent on the incubation time [36]. Considering that no additional mitochondrial or NADPHOX inhibitors were used in our experiments, it remains unclear if current AICAR-driven oxidative responses in bovine PMN correspond to NADPHOX-related ROS production or mitochondrial activities. Moreover, it remains to be elucidated if these differences indeed mirror host species-specific reactions, but, noteworthy, it is well documented that bovine PMN differ significantly from human PMN in their ROS-based responses to general stimulants [61]. In this regard, the differences and similarities of both host systems were recently reviewed [62] and call for more detailed analyses in the bovine system. Considering that we recently reported PMN clustering around *B. besnoiti* tachyzoites [24], it is tempting to speculate that chemotaxis and clustering itself are also regulated by AMPK activities in *B. besnoiti*-exposed PMNs; however, this hypothesis awaits further exploration in the future.

## 4. Materials and Methods

### 4.1. Ethics Statement

The current study was performed in accordance with the Justus Liebig University Giessen Animal Care Committee Guidelines. Protocols were approved by the Ethics Commission for Experimental Animal Studies of the Federal State of Hesse (Regierungspräsidium Giessen; GI 18/10 Nr. V 2/2022; JLU-No. 0002_V) and are in accordance with European Animal Welfare Legislation: ART13TFEU, and currently applicable German Animal Protection Laws.

### 4.2. Bovine PMN Isolation

Peripheral blood was collected by puncturing the jugular vein from healthy adult dairy cows and collected in heparinized sterile plastic tubes (Kabe Labortechnik, Nümbrecht, Germany). Then, 20 mL of heparinized blood was mixed with 20 mL of sterile PBS containing 0.02% EDTA (Carl Roth, Karlsruhe, Germany) and carefully layered on top of 12 mL of Histopaque-1077 separation solution (density = 1.077 g/L; 10771, Sigma-Aldrich, UK) and centrifuged (800× *g*, 45 min, RT) without brake. After removal of plasma and the buffy coat containing peripheral blood mononuclear cells, the cell pellet was suspended in 20 mL of lysis buffer (5.5 mM NaH_2_PO_4_ and 10.8 mM KH_2_PO_4_; pH 7.2) and gently mixed for 60 s to lyse erythrocytes. Osmolarity was rapidly restored by the addition of 10 mL of hypertonic buffer (462 mM NaCl, 5.5 mM NaH_2_PO_4_, and 10.8 mM KH_2_PO_4_; pH 7.2) and 10 mL of Hank’s balanced salt solution (HBSS, 14065-049, Gibco, Paisley, UK). The lysis step was repeated twice until no erythrocytes were present. PMN were then suspended in 5 mL of HBSS, counted in a Neubauer chamber, and allowed to rest on ice for 30 min before any experimental use.

### 4.3. Host Cell Culture and B. besnoiti Tachyzoite Maintenance

All parasite-related experiments of the current study were performed with tachyzoite stages of the apicomplexan parasite *B. besnoiti* (strain Bb-Evora04) initially isolated in Portugal. Madin–Darby bovine kidney (MDBK) cells were used as host cells for *B. besnoiti* tachyzoite in vitro production. MDBK cell layers were cultured in 75 cm^2^ plastic tissue culture flasks (658175, Greiner, Frickenhausen, Germany) at 37 °C and 5% CO_2_ atmosphere using RPMI 1640 (R7509, Sigma-Aldrich, UK) cell culture medium, supplemented with 5% fetal bovine serum (FBS; 10270-106, Gibco, Paisley, UK) and 1% penicillin/streptomycin (both 500 mg/mL, P4333, Sigma-Aldrich, Israel). MDBK cell layers were infected at 80% confluency with 2.4 × 10^7^ *B. besnoiti* tachyzoites. At 48–72 h post-infection (p. i.), newly formed tachyzoites were collected from cell supernatants, filtered through a 5 μm syringe filter (Merck Millipore, Burlington, NJ, USA), washed, and pelleted (400× *g*, 12 min) prior to re-suspension in RPMI 1640 cell culture medium. Tachyzoite numbers were determined in a Neubauer chamber, and tachyzoites were placed at 37 °C and 5% CO_2_ atmosphere until further experimental use.

### 4.4. Protein Extraction and Western Blot

Proteins from tachyzoite-exposed and non-exposed bovine PMN were extracted in RIPA buffer (50 mM Tris-HCl, pH 7.4; 1% NP-40; 0.5% Na-deoxycholate; 0.1% SDS; 150 mM NaCl; 2 mM EDTA; 50 mM NaF; all Roth, Karlsruhe, Germany) supplemented with a protease inhibitor cocktail (Sigma-Aldrich) by lysing 5 × 10^6^ PMN using an ultrasound sonicator (20 s, 5 cycles). Then, the samples were centrifuged (10,000× *g*, 10 min, 4 °C) to sediment intact cells and nuclei. Supernatants were collected and their protein content was quantified via the Pierce™ Bradford Plus Protein Assay Kit (23236, Thermo Scientific, Rockford, IL, USA) according to the manufacturer’s instructions. For immunoblotting, samples were supplemented with 6 M urea. After boiling (95 °C, 5 min), 40 μg of total protein was electrophoresed per slot in 12% or 15% polyacrylamide gels (100 V, 90 min) using a Mini-PROTEAN Tetra Cell system (Biorad, Feldkirchen, Germany). Proteins were then transferred (300 mA, 2 h) to polyvinylidene difluoride (PVDF) membranes (Millipore, Darmstadt, Germany) using a semidry blotting instrument (Mini-transfer blot, Biorad, Feldkirchen, Germany). The blots were first incubated in blocking solution (3% BSA in TBS containing 0.1% Tween, all Sigma-Aldrich; 1 h, RT) and then reacted overnight at 4 °C with primary antibodies (anti-AMPKα (Cat#50081 1:1000, Cell Signaling, Leiden, The Netherlands), anti-AMPKα Thr172 (Cat#5831, 1:1000, Cell Signaling, Leiden, The Netherlands), anti-pAMPKα (Cat#2795, 1:1000, Cell Signaling, Leiden, The Netherlands), anti-pAMPKβ1 (Cat#4178, 1:1000, Cell Signaling, Leiden, The Netherlands), anti-pAMPKγ1 (Cat#4187, 1:1000, Cell Signaling, Leiden, The Netherlands), anti-CAMKK (Cat#ab96531, 1:1000 Abcam, Cambridge, UK), anti-pCAMKK (Cat#abPA5-64569, 1:1000 Thermo Fischer), anti-Beclin-1 (Cat#3495, 1:1000 Cell Signaling, Leiden, The Netherlands), anti-p-Beclin-1 (Cat#14717, 1:1000 Cell Signaling, Leiden, The Netherlands), and anti-ULK1 (Cat#8054, 1:1000 Cell Signaling, Leiden, The Netherlands)) diluted in blocking solution. The detection of vinculin (Cat#sc-73614, 1:1000, Santa Cruz, Texas, LA, USA) was used for normalization of the samples. Signal detection was accomplished by incubation for 30 min at RT in the corresponding secondary antibodies conjugated with peroxidase (Cat#31430, 1:40,000 and Cat#31460, 1:40,000, both Pierce) and then applying an enhanced chemiluminescence detection system (ECL^®^ plus kit, RPN2132, GE Healthcare, Buckinghamshire, UK). Protein signals were recorded in a ChemoCam Imager^®^ (Biorad, Feldkirchen, Germany). Protein masses were controlled by a protein ladder (PageRuler^®^plus pre-stained protein ladder covering ~10–250 kDa; Thermo Fisher Scientific, Rockford, IL, USA). Quantification of protein band intensities was performed by Image J^®^ software (Fiji version using the gel analyzer plugin).

### 4.5. Quantification of Neutrophil Oxygen Consumption and Extracellular Acidification Rates

Activation of bovine PMN was monitored on the level of oxidative and glycolytic responses using the Seahorse XF analyzer (Agilent, Santa Clara, CA, USA). Briefly, 1 × 10^6^ PMN from three blood donors were pelleted at 500× *g* (10 min, room temperature (RT)) and resuspended in 0.25 mL of XF assay medium (Agilent, Santa Clara, CA, USA) supplemented with 2 mM of _L_-glutamine, 1 mM of pyruvate, and 10 mM of glucose. A total of 2 × 10^5^ cells were gently placed in each well of an eight-well XF analyzer plate (Agilent, Santa Clara, CA, USA) pre-coated for 30 min with 0.001% poly-_L_-lysine (Sigma-Aldrich, P8920, St. Louis, MI, USA). XF assay medium (Agilent) was adjusted to a 180 μL total volume per well and cells were incubated at 37 °C without CO_2_ supplementation for 45 min before Seahorse measurements. AICAR (10 mM) and *B. besnoiti* tachyzoites (1.2 × 10^6^/well) were suspended in XF assay medium and supplemented with PMN via instrument-own injection ports after baseline measurements. The total assay duration was 240 min. Background subtraction and determination of oxygen consumption rate (OCR) and extracellular acidification rate (ECAR) registries were analyzed using the Seahorse Agilent analytics platform (https://seahorseanalytics.agilent.com).

### 4.6. Analysis of Neutrophil Apoptosis and Necrosis by Flow Cytometry

Apoptosis and necrosis rates in AICAR- and compound-C-treated PMN were determined by a commercial cell viability test kit (Cat# ab14085, Abcam, Cambridge, UK), which was based on Annexin-V and propidium iodide (PI) staining, following the manufacturer instructions.

### 4.7. Immunofluorescence-Based Detection and Quantification of NET Formation

Unstimulated bovine PMN (negative control) and PMN incubated with *B. besnoiti* tachyzoites (1:4) in the presence or absence of 1 mM of AICAR were fixed after 4 h of co-culture (37 °C, 5% CO_2_) with 4% paraformaldehyde (15 min, RT). Bovine PMN incubated in RPMI plain medium were used as negative controls. After incubation, the samples were carefully washed thrice with sterile PBS and incubated in blocking/permeabilization solution (PBS containing 3% BSA, 0.3% Triton X-100; Sigma-Aldrich, St. Louis, MI, USA) for 1 h at RT. Then, samples were reacted with primary antibodies (anti-histone-DNA, 1:200 Cat# MAB3864, Merck-Millipore, Darmstadt, Germany; anti-neutrophil elastase, NE, Cat# ab6872, Abcam, Cambridge, UK) diluted in blocking/permeabilization solution (overnight, 4 °C, humidified chamber). Thereafter, samples were washed thrice in sterile PBS and incubated for 30 min at RT, protected from light, with corresponding secondary antibodies (anti-rabbit IgG Alexa 488, 1:500, Cat# A11008, and anti-mouse IgG Alexa 594, Cat# A11005, both Thermo Fisher, Eugene, ON, USA). DNA counterstaining was accomplished by 4′,6-diamidin-2-phenylindol (DAPI) present in mounting medium (Fluoromount G, 00-4959-52, Thermo Fisher, Waltham, MA, USA). Images were acquired by a Nikon Eclipse Ti2-A inverted microscope equipped with ReScan confocal microscopic instrumentation (RCM 1.1 Visible, Confocal.nl) and a motorized z-stage (DI1500). Three channels were recorded for signal detection: DAPI/Blue/405-laser, AlexaFluor488/Green/Argon-laser, and AlexaFluor594/Red/HeNe-543-laser. Images were acquired by a sCMOS camera (PCO edge) using a CFI Plan Apochromat X60 lambda-immersion oil objective (NA 1.4/0.13; Nikon) controlled either by Zeiss ZEN 2010 or by NIS-Elements v 5.11 (Nikon, Tokyo, Japan) software. Samples were imaged via z-stack optical series with a step size of 0.2–0.3 microns in depth. The z-series were displayed as maximum z-projections, and all settings (gamma, brightness, and contrast) were applied at identical conditions when comparing image sets using Image J software, Fiji version [63]. Measurements of defined parameters (e.g., area, integrated density, and number) were performed with Fiji/Image J software (version: 1.53c) [63]. Histone-DNA and DAPI signals were acquired at the same time point for each image. A manual threshold was applied to each channel using the clustering algorithm of Otsu [64]. Sharpness of the images was adjusted and the percentage of cells releasing NETs for each experimental condition was assessed, as described by Brinkmann et al. [37].

### 4.8. Statistical Analysis

Statistical significance was defined by a *p*-value < 0.05. In the Western blot experiments, the *p*-values were calculated via unpaired, two-tailed *t*-tests, comparing control PMN vs. PMN incubated with *B. besnoiti* tachyzoites at each time point. For Seahorse-based metabolic measurements, the *p*-values were calculated using the Mann–Whitney test. Kruskal–Wallis followed by Dunn’s multiple comparisons tests were applied to NET quantification results. Bar graphs represent the mean ± SD, and statistical analysis was performed by GraphPad software (v. 7.03).

## Data Availability

The raw data supporting the conclusions of this article will be made available by the authors upon request.
